# Author Correction: The protein arginine methyltransferase PRMT9 attenuates MAVS activation through arginine methylation

**DOI:** 10.1038/s41467-023-44545-9

**Published:** 2024-01-04

**Authors:** Xuemei Bai, Chao Sui, Feng Liu, Tian Chen, Lei Zhang, Yi Zheng, Bingyu Liu, Chengjiang Gao

**Affiliations:** 1 Key Laboratory of Infection and Immunity of Shandong Province & Department of Immunology, School of Basic Medical Sciences, Shandong University, Jinan, Shandong 250012 PR China; 2 Department of Pathogenic Biology, School of Biomedical Sciences, Shandong University, Jinan, Shandong 250012 PR China

**Keywords:** Infection, Autoimmunity, RIG-I-like receptors, Innate immunity

Correction to: *Nature Communications* 10.1038/s41467-022-32628-y, published online 26 August 2022

The original version of Figure 4e contained an error, in which the Actin gel image was mistakenly replaced with the TBK1 gel image from the same experiment; the uncropped images in the Source Data file associated with this panel was correct.

In addition, there is a textual error in page 2, in which the sentence “Further, we also observed that the colocalization of the endogenous PRMT9 with mitochondria was attenuated following the SeV infection in PM or THP-1 cells (Supplementary Fig. 2e, f)” incorrectly read “Further, we also observed that the colocalization of the endogenous PRMT9 with MAVS was attenuated following the SeV infection in PM or THP-1 cells (Supplementary Fig. 2e, f)”.

Lastly, there were several errors in the citation and reference list.

All the above errors have been corrected in both the PDF and HTML versions of the Article.

