# Supplementary material for: The protein arginine methyltransferase PRMT9 attenuates MAVS activation through arginine methylation
Source: Nat Commun. 2022 Aug 26;13:5016. doi: 10.1038/s41467-022-32628-y (PMC9418238; doi:10.1038/s41467-022-32628-y)
Supplement: Supplementary file 1 — Supplementary information [file 41467_2022_32628_MOESM1_ESM.pdf]

Supplementary Information

The protein arginine methyltransferase PRMT9 attenuates MAVS activation through symmetric arginine methylation

Xuemei Bai[1], Chao Sui[1], Feng Liu[1], Tian Chen[2], Lei Zhang[1], Yi Zheng[1], Bingyu Liu[1*], Chengjiang Gao[1,2*]

[1]Key Laboratory of Infection and Immunity of Shandong Province & Department of Immunology, School of Biomedical Sciences, Shandong University, Jinan, Shandong 250012, P. R. China

[2]Lead Contact

[*]Correspondence: Dr. Chengjiang Gao

E-mail: cgao@sdu.edu.cn

Tel: (86)531-88382292

Fax: (86)531-88382292

Or Dr. Bingyu Liu

E-mail: liubingyu@sdu.edu.cn

Supplementary Figure 1-9

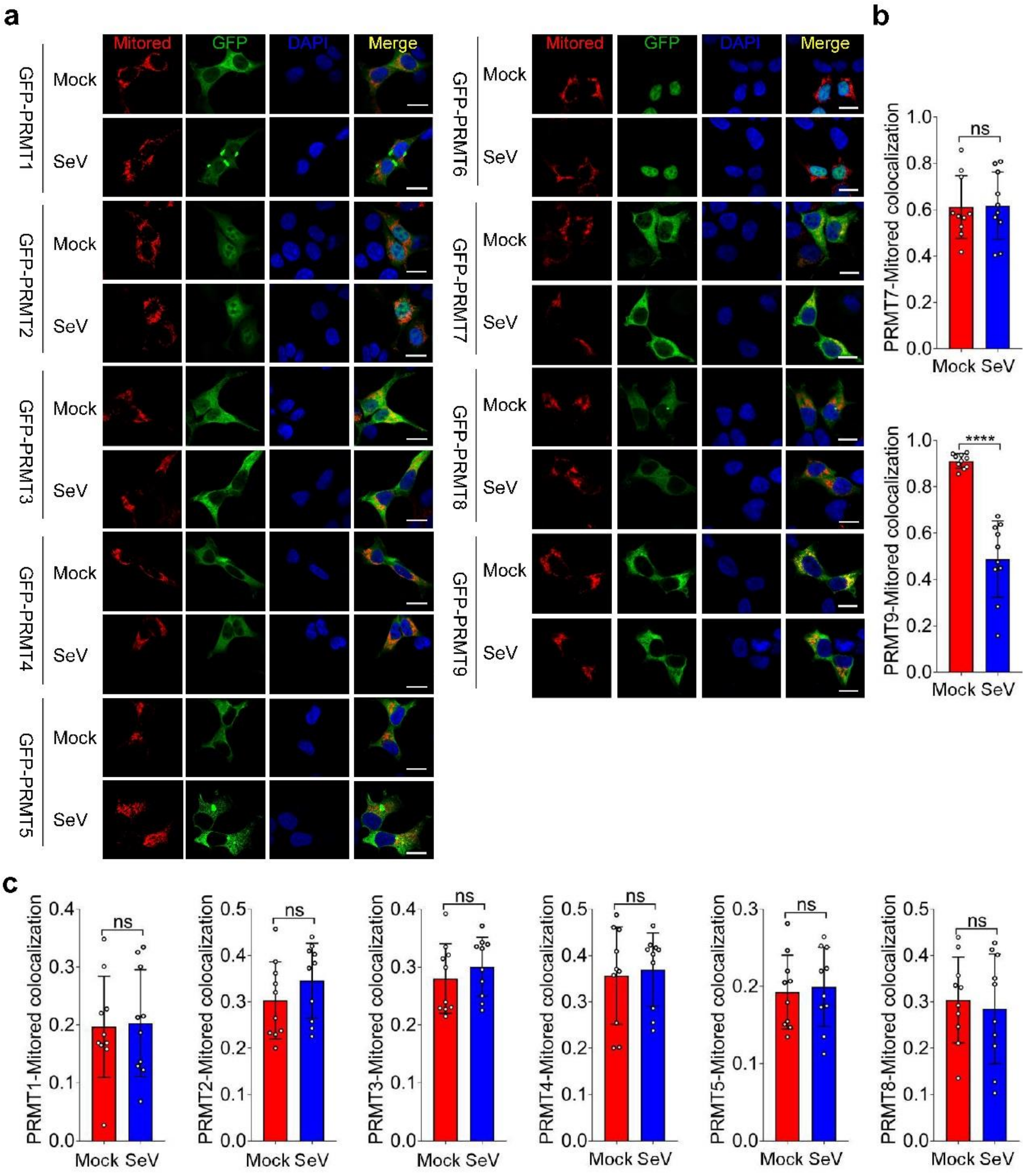

Supplementary Figure 1. PRMT9 localizes in mitochondrial

a) Confocal analysis were used to measure the mitochondrial localization of GFP-PRMT1-9 in HEK293T cells with SeV infection or not. HEK293T cells cotransfected with GFP-PRMTs and DsRed2-Mitored for 24 h, followed by infection with SeV for 8h, Scale bars: 5 μm (n = 3 independent experiments). **b**, **c**) Image J software was used to quantitatively analyze the colocalization between PRMTs with mitochondria in HEK293T cells. Cells expressing both PRMTs and DsRED2-Mitored were selected

randomly for colocalization analysis. Pearson’s Coefficient was quantified by Scatter J, and drawn by GraphPad Prism 7.0. Quantification of immunostaining data in (**b**, **c**) represent the mean ± SD (n = 10 cells per group). For **b**: ns = 0.9253, ****P < 0.0001; for **c** ns = 0.8799, 0.2593, 0.4301, 0.7575, 0.7620, 0.6959 in sequence. ns, not significant (two-tailed Student's *t*-test).

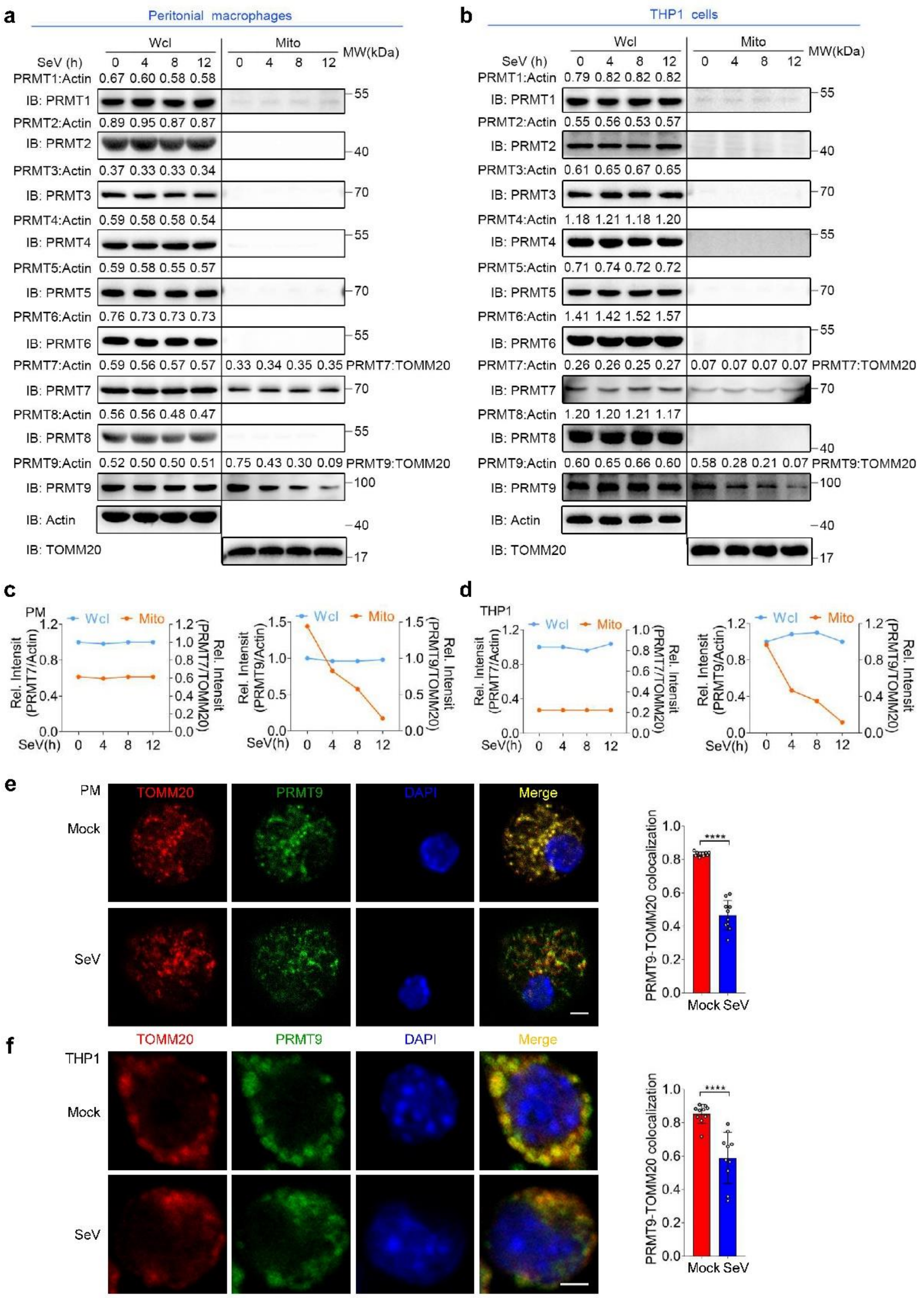

Supplementary Figure 2. **PRMT9 colocalized with the mitochondria**

a, **b**) Immunoblot analysis of the expression of PRMTs in total cell lysates or crude mitochondria lysates from peritoneal macrophages (PM) or THP1 cells infected with SeV for 0-12 h.

c, **d**) Immunoblot was quantified by Image J software (PRMTs were normalized with individual actin or tomm20), and the results were presented relative to lane 1 both in total cell lysates and crude mitochondria lysates.

e, **f**) Confocal microscope imaging was used to measure the colocalization of the endogenous PRMT9 (Green) with mitochondria (Red) in PM (**e**) and THP1 cells (**f**) infected with SeV for 0-8 h. Scale bars: 2 μm. PRMT9-TOMM20 colocalization was quantified using Pearson's correlation coefficient method, and drawn by GraphPad Prism 7.0. Quantification of immunostaining data in (**e**, **f**) represent the mean ± SD (n = 10 cells per group). ****$P < 0.0001$ (two-tailed Student's *t*-test). The experiments were performed for at least two times with similar results.

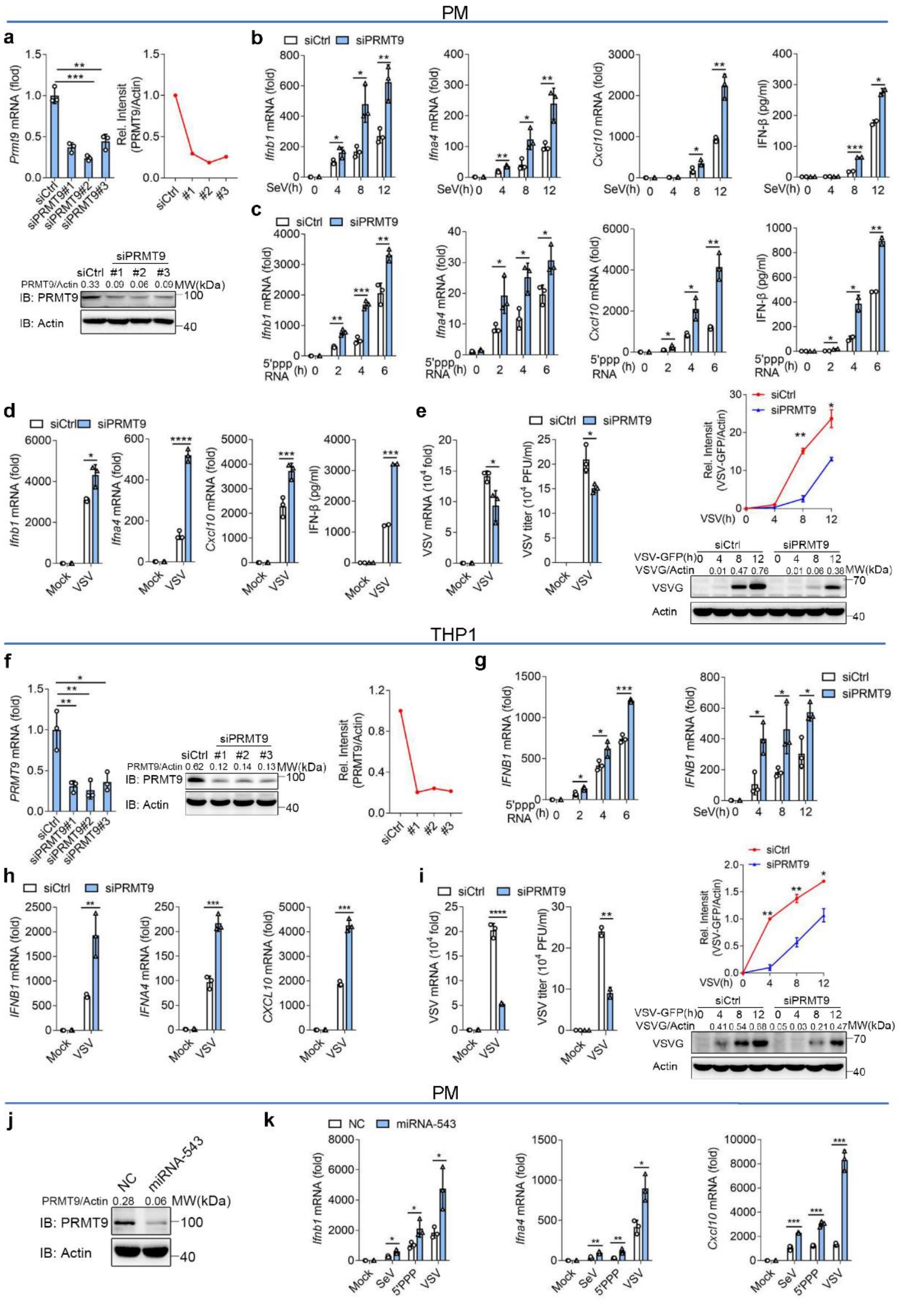

Supplementary Figure 3. PRMT9 negatively regulates RLRs-induced IFN-β signaling

a) qRT-PCR analysis of *Prmt9* mRNA expression and immunoblot analysis of PRMT9

protein expression (down) in mouse primary peritoneal macrophages transfected with control siRNA (siCtrl) or siRNA targeting PRMT9 (siPRMT9#1, ***P = 0.0006; siPRMT9#2, ***P = 0.0002 or siPRMT9#3, **P = 0.0018) for 48 h. **b-d**) qRT-PCR analysis the expression levels of *Ifnb1*, *Ifna4* or *Cxcl10* mRNA and ELISA analysis the production of IFN-β protein (far right) in primary peritoneal macrophages transfected with control siRNA (siCtrl) or mice PRMT9 siRNA (siPRMT9) for 48 h, followed by infection with SeV, VSV or stimulated with 5′-pppRNA. **e**) qRT-PCR analysis the expression of VSV mRNA (left), plaque assay of VSV titers (middle) and immunoblot analysis of VSV glycoprotein (VSV-G) (right) in peritoneal macrophages. For the densitometric analysis (right), VSV bands were normalized with individual actin, line graphs were presented relative to the second lane. (Representative data were collected and expressed as mean ± SD from three independent experiments. Two-tailed Student's t test siPRMT9 vs. siCtrl, for **b**, *Ifnb1*: *p = 0.0404, *p = 0.0121, **p = 0.0078 in sequence; *Ifna4*: **p = 0.0023, *p = 0.0197, **p = 0.0093 in sequence; *Cxcl10*: *p = 0.0347, **p = 0.0015 in sequence; IFN-β (n = 2 independent samples) : ***p = 0.0003, *p = 0.0106 in sequence. For **c**, *Ifnb1*: **p = 0.0013, ***p = 0.0003, **p = 0.0049 in sequence; *Ifna4*: *p = 0.0355, 0.0144, 0.0255 in sequence; *Cxcl10*: *p = 0.0264, 0.0151 , **p = 0.0015 in sequence; IFN-β (n = 2 independent samples) : *p = 0.0299, *p = 0.0316, **p = 0.0043 in sequence. For **d**, *p = 0.0156, ****$P < 0.0001$, ***p = 0.0080; IFN-β (n = 2 independent samples): ***p = 0.0001 in sequence. For **e**, VSV mRNA: *p = 0.0319; VSV titier : *p = 0.0244; VSV-GFP/Actin (n = 2 independent samples): **p = 0.0038, *p = 0.0245). **f**) qRT-PCR analysis of *PRMT9* mRNA expression and immunoblot analysis of PRMT9 protein expression in THPI cells as in (**a**). **g**) qRT-PCR analysis the expression of *IFNB1* in THP1 cell infection with SeV or stimulated with 5′-pppRNA. **h**) qRT-PCR analysis of *IFNB1*, *IFNA4*, *CXCL10* mRNA in THP1 cells treated as in (**d**). **i**) Immunoblot analysis of VSV glycoprotein (VSV-G) (right) in peritoneal macrophages in THP-1 cells infected with VSV (MOI, 0.1). For the densitometric analysis (right), VSV peoteins were normalized with individual actin, line graphs were presented relative to the second lane. (Representative data were collected and expressed as mean ± SD from three independent experiments. Two-tailed

Student’s t test siPRMT9 vs. siCtrl, for **f**, **p = 0.0086 (#1), **p = 0.0083 (#2), *p = 0.0141(#3); for **g**, left panel: *p = 0.0177, *p = 0.0299, ***p = 0.0001 in sequence; right panel: *p = 0.0145, 0.0395, 0.0143 in sequence; for **h**, **p = 0.0080; ***p = 0.0006; ***p = 0.0001 in sequence; for **i**, VSV mRNA: **** $P < 0.0001$; VSV titier: **p = 0.0088; VSV-GFP/Actin (n = 2 independent samples): **p = 0.0018, **p = 0.0097, *p = 0.0186). **j**) Immunoblot analysis of PRMT9 protein expression in mouse primary peritoneal macrophages transfected with control RNA (NC) or miRNA-543. **k**) qRT-PCR analysis the expression levels of *Ifnb1*, *Ifna4* or *Cxcl10* mRNA in primary peritoneal macrophages transfected with control RNA (NC) or miRNA-543 for 48 h, followed by infection with SeV, VSV or stimulated with 5′-pppRNA. (Representative data were collected and expressed as mean ± SD from three independent experiments. Two-tailed Student’s t test miRNA-543 vs. NC , for **k**, left panel: *p = 0.0160, 0.0349, 0.0252 in sequence; middle panel: **p = 0.0052, **p = 0.0079, *p = 0.0112 in sequence; right panel: ***p = 0.0005, 0.0002, 0.0001 in sequence). The qRT-PCR and ELISA results are presented relative to those of untreated cells transfected with control siRNA (Average of three replicates, **a**-**i** and **k**). *$P < 0.05$; **$P < 0.01$; ***$P < 0.001$; ****$P < 0.0001$ (two-tailed Student's *t*-test).

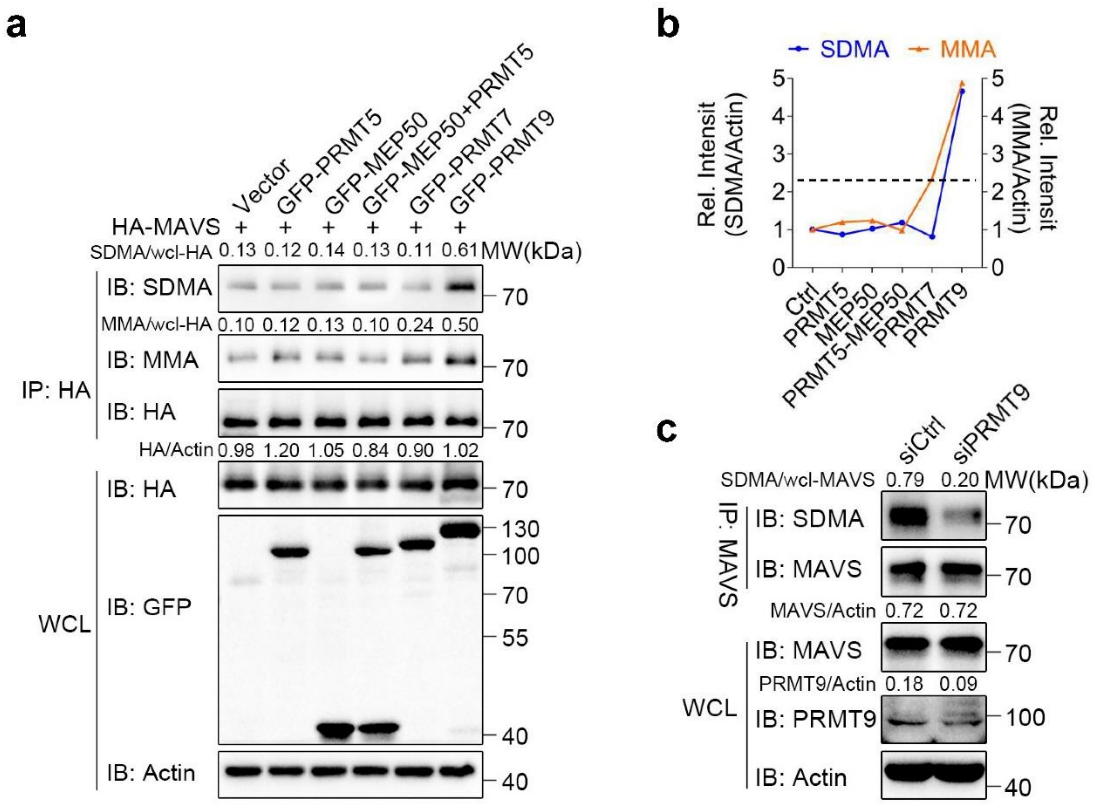

Supplementary Figure 4. PRMT9 promotes MAVS methylation

a, **b**) Co-IP analysis of the methylation of MAVS in HEK293T cells cotransfected with GFP-PRMT9, GFP-PRMT5, GFP-MEP50, GFP-PRMT7 and HA-MAVS. **b**) Densitometric analysis of protein expression levels. Ratio: SDMA/wcl-HA, MMA/wcl-HA, line graphs are presented relative to the lane 1. **c**) Co-IP analysis of the methylation of MAVS in mouse peritoneal macrophages transfected with control siRNA (siCtrl) or siRNA targeting PRMT9 transfected for 48 h.

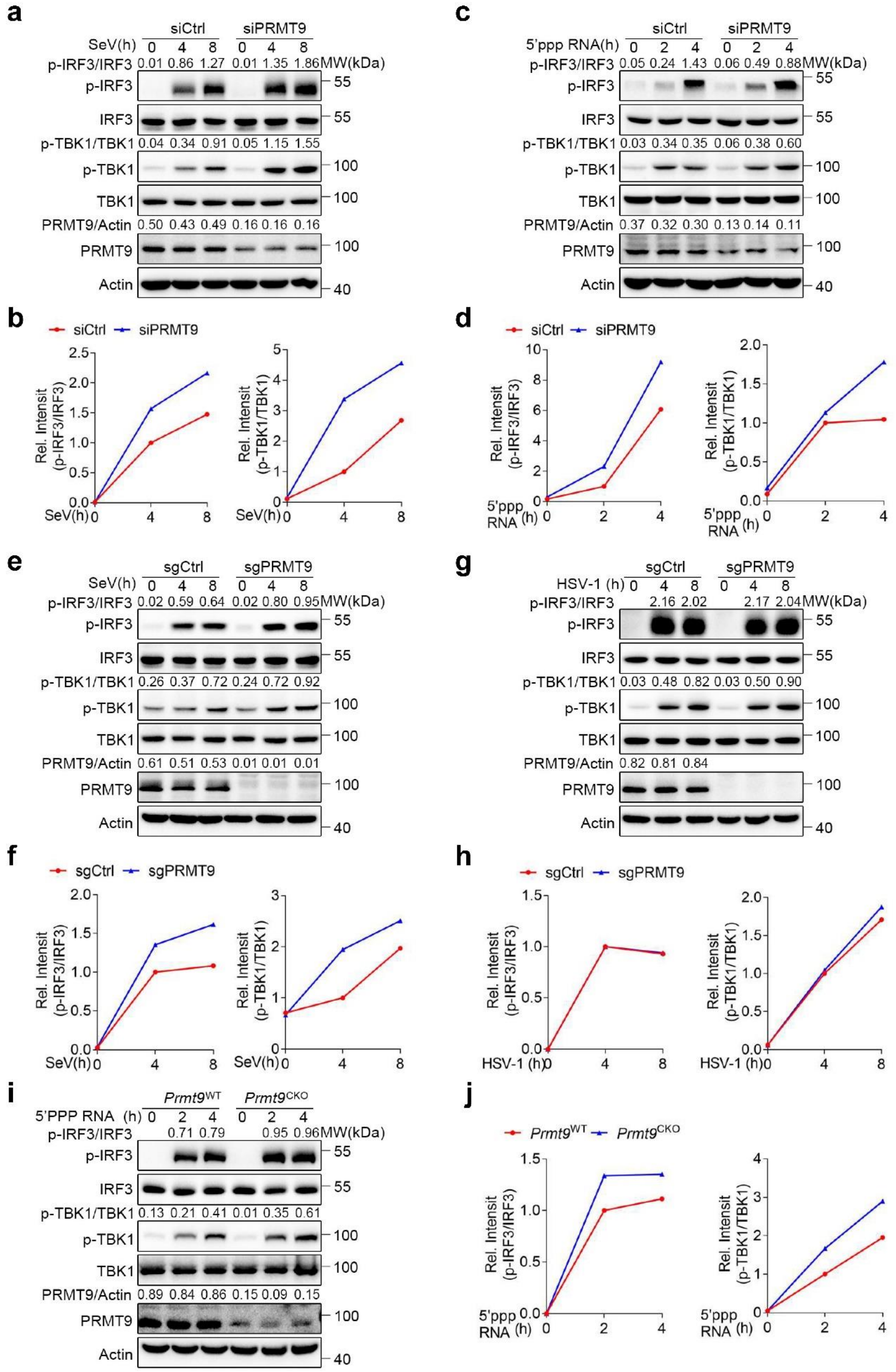

Supplementary Figure 5. PRMT9 regulates phosphorylation of TBK1 and IRF3

a, **c**) Immunoblot analysis of total and phosphorylated (p-) TBK1, total and

phosphorylated (p-) IRF3 in peritoneal macrophages cells, followed by infection with SeV or stimulated with 5′-pppRNA. **b**, **d**) Densitometric analysis of protein expression levels. Ratio: p-TBK1/TBK1, p-IRF3/IRF3 in mouse PMs infection with SeV or stimulated with 5′-pppRNA, line graphs are presented relative to the second lane. **e**, **g**) Immunoblot analysis of total and phosphorylated (p-) TBK1, total and phosphorylated (p-) IRF3 in *Prmt9*-knockout cell lines in RAW264.7 cells, followed by infection with SeV or HSV-1. **f**, **h**) Densitometric analysis of protein expression levels. Ratio: p-TBK1/TBK1, p-IRF3/IRF3 in *Prmt9*-knockout cell lines in RAW264.7 cells infection with SeV or HSV-1, line graphs are presented relative to the second lane. **i**) Immunoblot analysis of p-TBK1 and p-IRF3, in *Prmt9*^{CKO} and *Prmt9*^{WT} peritoneal macrophages cells, followed by infection with 5′-pppRNA. **j**) Densitometric analysis of protein expression levels. Ratio: p-TBK1/TBK1, p-IRF3/IRF3, results are presented as in (**b**). The data shown in **a**, **c**, **e**, **g** and **i** are from one representative experiment of at least 2 biological independent experiments.

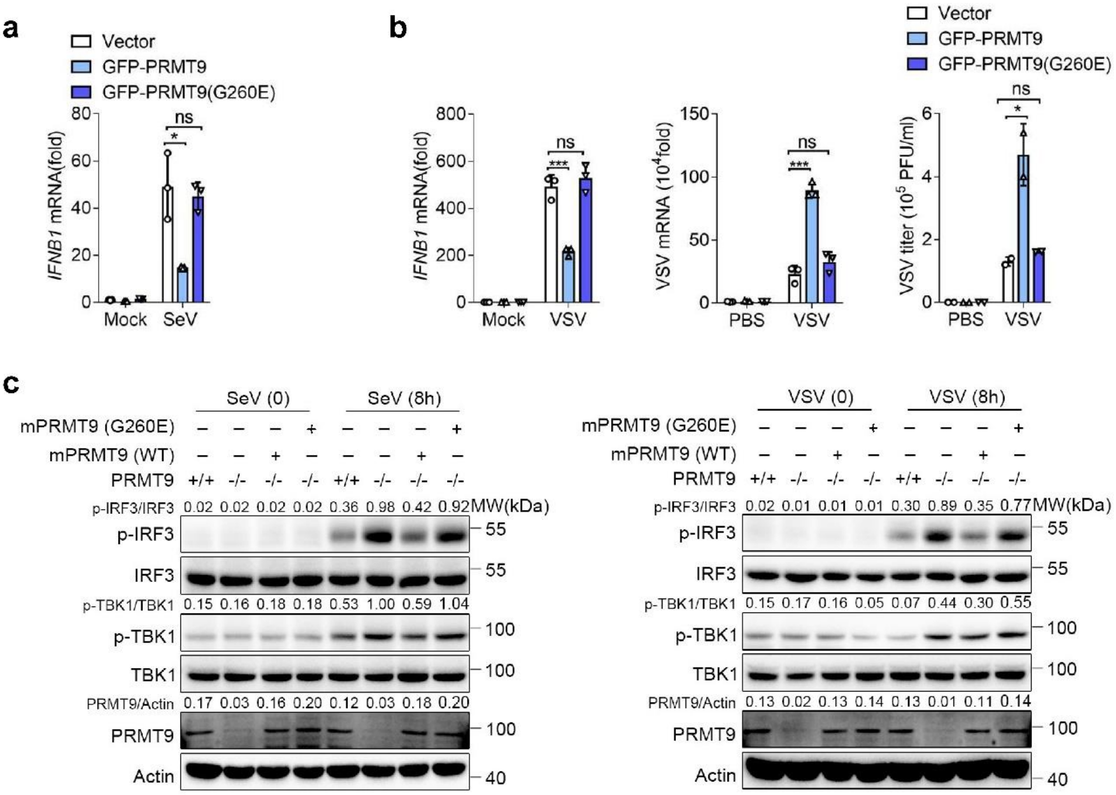

Supplementary Figure 6. PRMT9-mediated MAVS activation depends on its methyltransferase activity.

a) qRT-PCR analysis of *IFNB1* in HEK293T cells transfected with GFP-PRMT9 and PRMT9 (G260E), followed by infection with SeV for 8 h, mRNA results are presented relative to those of untreated cells transfected with Vector plasmid (Average of three replicates). **b**) qRT-PCR analysis of *IFNB1* (left), VSV mRNA (middle) and plaque assay of VSV titers (right) in HEK293T cells transfected as in (**a**), infected with VSV (MOI, 0.1) for 8 h, mRNA results are presented as in (**a**). Two-tailed Student's t test was performed, for **a**, *$p = 0.0136$, ns = 0.6626; for **b**, left panel: ***$p = 0.0008$, ns = 0.4582; middle panel: ***$p = 0.0002$, ns = 0.1879; left panel: *$p = 0.0406$, ns = 0.0910. **c**) Immunoblot analysis of p-TBK1, p-IRF3 in *Prmt9*^{CKO} and *Prmt9*^{WT} peritoneal macrophages cells reconstituted with empty vector or plasmid mPRMT9 (WT) or mPRMT9 (G260E), followed by infection with SeV or VSV, for 8h. RT-qPCR data in (**a**, **b**) represent the mean ± SD (n = 3 independent experiments). The data shown in **c** are from one representative experiment of at least two biological independent experiments. Two-tailed Student's t test was performed, with *$P < 0.05$; ***$P < 0.001$;

ns, not significant (two-tailed Student's t-test).

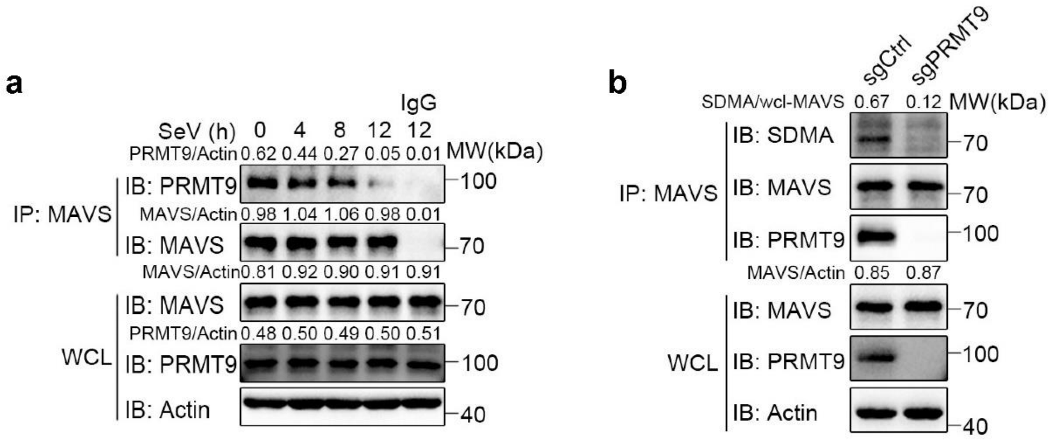

Supplementary Figure 7. PRMT9 interacts with MAVS and catalyzes MAVS SDMA

a) Co-IP analysis of the interaction between PRMT9 and MAVS in RAW264.7 cells, followed by infection with SeV for 0-12 h. Densitometric analysis of protein expression levels, bands were normalized with individual actin.

b) Co-IP analysis of the methylation of MAVS in *Prmt9*-knockout cell lines in RAW264.7 cells, and for the densitometric analysis, bands were normalized with individual target protein.

Similar results were obtained from least two independent experiments.

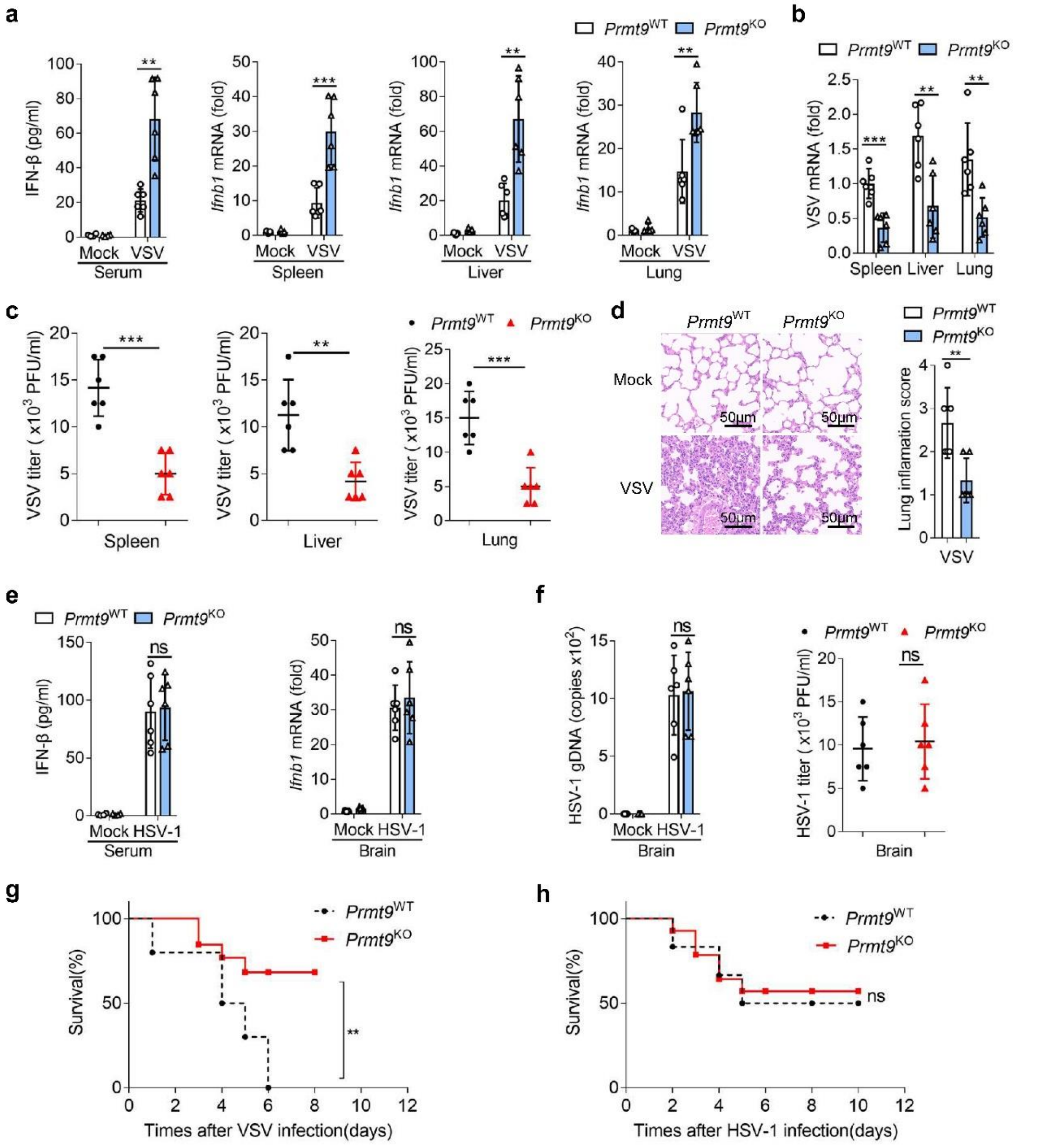

Supplementary Figure 8. PRMT9 deficiency attenuates VSV infection *in vivo*

Cre^{ERT} *Prmt9*$^{fl/fl}$ mice (call *Prmt9*^{KO} here) were infected by tail vein injection with VSV (1.8×10^7 PFU per mouse, n = 6 mice per group) for 24 h. **a**) ELISA analysis of IFN-β protein in serum (***p* = 0.0010), and qRT-PCR analysis of *Ifnb1* mRNA in the spleen (****p* = 0.0007), liver (***p* = 0.0015), and lung (***p* = 0.0079). Plaque assay of VSV titers (**c**) and qRT-PCR analysis of VSV mRNA (**b**) in lungs, liver, and spleen. Two-tailed Student's t test was performed, for **b**, ****p* = 0.0004, ***p* = 0.0033,***p* = 0.0063 in sequence; for **c** ****p* = 0.0001, ***p* = 0.0024, ****p* = 0.0004 in sequence. **d**)

Hematoxylin-eosin staining of lung sections were presented, treated as in (**a**). Scale bar, 50 μm, (n = 6 mice per group, $^{**}p = 0.0070$). Inflammation scores of lung tissue sections described in d.

e, **f**) *Prmt9*^{KO} and *Prmt9*^{WT} mice were injected with HSV-1 (1.5×10^8 PFU per mouse) for 24 h by tail vein injection (n = 6 mice per group). **e**) ELISA analysis of IFN-β protein in serum, and qRT-PCR analysis of *Ifnb1* mRNA in brains. **f**) qRT-PCR analysis the copy number of HSV-1 genomic DNA and plaque assay of viral titier of HSV-1 in brains. **g**, **h**) Survival of *Prmt9*^{KO} and *Prmt9*^{WT} mice (n = 12 mice per group, 6-8 weeks old) after tail vein injection with VSV (1×10^8 PFU per mouse) or HSV-1 (1.5×10^8 PFU per mouse). The qRT-PCR and ELISA results are presented relative to those of untreated wild-type tissue cells (**a**, **b**, **e** and **f**). Data are shown as mean ± SD.(**a-f**) and are representative of three independent experiments with similar results. $^{*}P < 0.05$; $^{**}P < 0.01$; $^{***}P < 0.001$ (two-tailed Student's *t*-test in **a-f**). The log-rank Mantel–Cox test in **g** ($^{**}P = 0.0049$), **h** (ns = 0.7923).

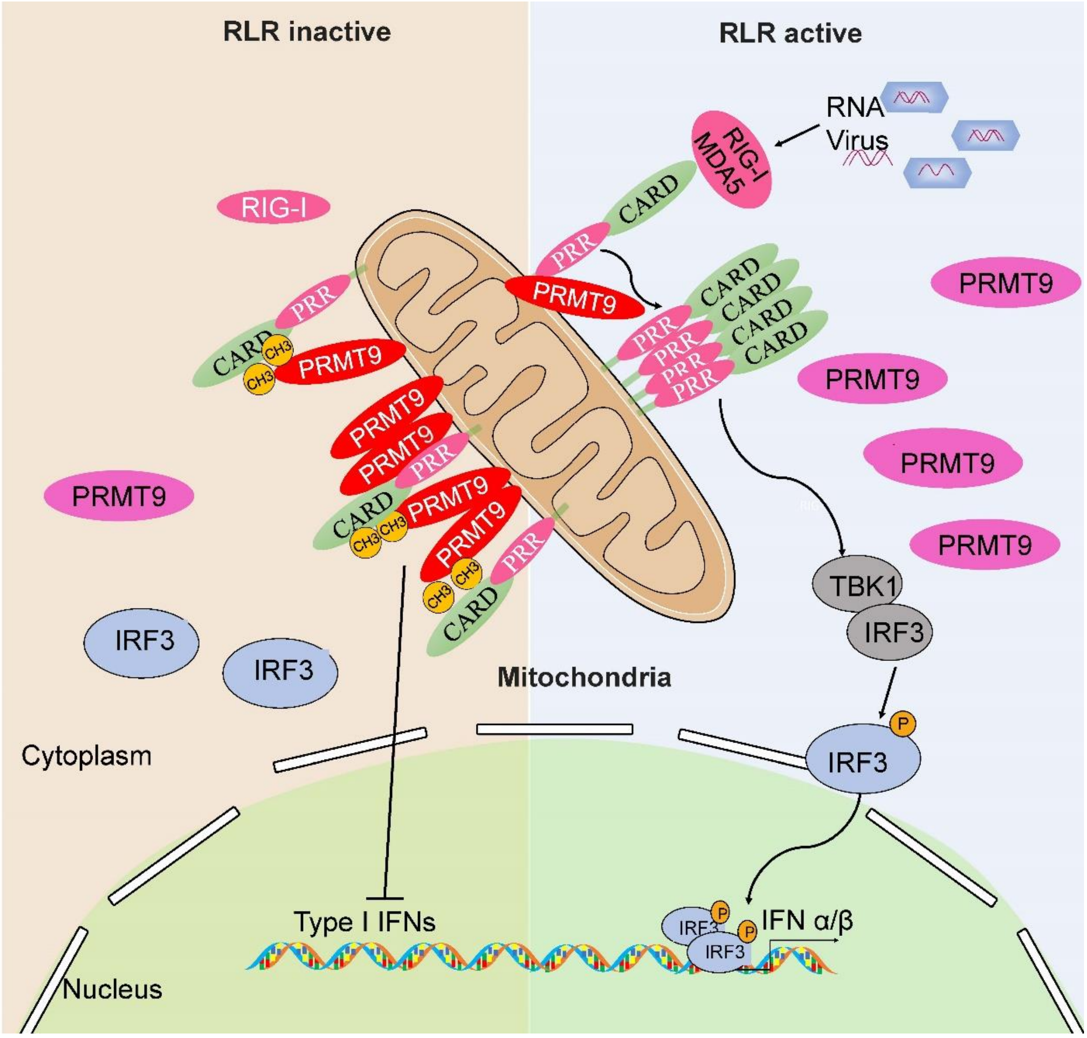

Supplementary Figure 9. Schematic model to show PRMT9 attenuates MAVS activation through arginine methylation. PRMT9 targets MAVS directly and catalyzes the arginine methylation of MAVS. In the resting state, this modification inhibits MAVS aggregation and autoactivation of MAVS. Upon virus infection, PRMT9 is dissociated from the mitochondria, leading to the aggregation and activation of MAVS.

Supplementary Tables

Supplementary Table 1: Oligonucleotides used in this study

siRNAs		
Gene	species	siRNA (5'-3')
PRMT9	Mouse 1	GGAGAGCACCUCUUCAGAATT
	Mouse 2	CCAGAACCGUUCUGGAUAUTT
	Mouse 3	GGACAUUGCUGGUAUACAUTT
PRMT9	Human 1	GCUUAACAACAUCCCAUAUTT
	Human 2	GGUAUCCAUUUGCCAACAATT
	Human 3	CCUCAUAUCUGAAGCCAAUTT

**Supplementary Table 2: Primers for RT-qPCR**			
Gene	Species	Forward primer (5'-3')	Reverse primer (5'-3')
Ifnb1	Mouse	AGTTACACTGCCTTTGCC	GTTGAGGACATCTCCCAC
Ifna4	Mouse	ACCCACAGCCCAGAGAGTGAC<br>C	AGGCCCTCTTGTTCCCGAGGT
Cxcl10	Mouse	CCAAGTGCTGCCGTCATTTT	GATAGGCTCGCAGGGATGAT
Actin	Mouse	CCACACCCGCCACCAGTTCG	TACAGCCCGGGGAGCATCGT
Prmt9	Mouse	AGTTGGCCACATGGAGTTGA	AAAGTTGTATCAACAACCCTA<br>AACA
PRMT9	Human	AGCACATTCCGTGTATGCCT	ACTAGGGACACTCTTTCGGG
IFNB1	Human	CAACAAGTGTCTCCTCCAAAT	TCTCCTCAGGGATGTCAAAG
IFNA4	Human	GACTCATCTGCTGCTTG	AGGGCTGTATTTCTTCTC
CXCL10	Human	TGCCATTCTGATTTGCTGCC	TGATGGCCTTCGATTCTGGA
ACTIN	Human	GGAAATCGTGCGTGACATTAA	AGGAAGGAAGGCTGGAAGAG
VSV		ACGGCGTACTTCCAGATGG	CTCGGTTCAAGATCCAGGT
